# Detection of *Clostridium tetani* Neurotoxins Inhibited In Vivo by Botulinum Antitoxin B: Potential for Misleading Mouse Test Results in Food Controls

**DOI:** 10.3390/toxins10060248

**Published:** 2018-06-19

**Authors:** Luca Bano, Elena Tonon, Ilenia Drigo, Marco Pirazzini, Angela Guolo, Giovanni Farina, Fabrizio Agnoletti, Cesare Montecucco

**Affiliations:** 1 Istituto Zooprofilattico Sperimentale delle Venezie, 35020 Legnaro, Italy; etonon@izsvenezie.it (E.T.); idrigo@izsvenezie.it (I.D.); aguolo@izsvenezie.it (A.G.); gfarina@izsvenezie.it (G.F.); fagnoletti@izsvenezie.it (F.A.); 2 Department of Biomedical Sciences, University of Padova, 35131 Padua, Italy; marco.pirazzini@unipd.it (M.P.); cesare.montecucco@unipd.it (C.M.)

**Keywords:** mouse test, *Clostridium tetani*, botulinum antitoxin, food safety

## Abstract

The presence of botulinum neurotoxin-producing Clostridia (BPC) in food sources is a public health concern. In favorable environmental conditions, BPC can produce botulinum neurotoxins (BoNTs) outside or inside the vertebrate host, leading to intoxications or toxico-infectious forms of botulism, respectively. BPC in food are almost invariably detected either by PCR protocols targeted at the known neurotoxin-encoding genes, or by the mouse test to assay for the presence of BoNTs in the supernatants of enrichment broths inoculated with the tested food sample. The sample is considered positive for BPC when the supernatant contains toxic substances that are lethal to mice, heat-labile and neutralized in vivo by appropriate polyclonal antibodies raised against purified BoNTs of different serotypes. Here, we report the detection in a food sample of a *Clostridium tetani* strain that produces tetanus neurotoxins (TeNTs) with the above-mentioned characteristics: lethal for mice, heat-labile and neutralized by botulinum antitoxin type B. Notably, neutralization occurred with two different commercially available type B antitoxins, but not with type A, C, D, E and F antitoxins. Although TeNT and BoNT fold very similarly, evidence that antitoxin B antiserum can neutralize the neurotoxic effect of TeNT in vivo has not been documented before. The presence of *C. tetani* strains in food can produce misleading results in BPC detection using the mouse test.

## 1. Introduction

Botulism is a neuroparalytic illness caused by the action of heat-labile botulinum neurotoxins (BoNT) produced by Gram-positive, spore-forming, anaerobe microorganisms belonging to the genus *Clostridium*. Seven confirmed BoNT serotypes (types A–G) have been recognized, characterized by the continuous isolation of intratypic variants known as subtypes and indicated with an Arabic number (BoNT/A1, BoNT/A2, BoNT/B1, BoNT/B2, etc.) [1,2]. A potential eighth type (“type H”) was reported in 2013 and a new toxin, displaying the lowest degree of similarities among BoNTs, dubbed BoNT/X, has been recently described [3,4].

Botulism is characterized by the flaccid paralysis of cranial and skeletal muscles, possibly leading to death by respiratory failure [5]. The disease may occur following the intake of BoNTs preformed outside (intoxication) or produced inside the host (toxico-infection). The most frequent form of BoNT intoxication in humans is food-borne botulism due to consumption of food where BoNT-producing Clostridia (BPC) have found adequate conditions for spore germination and neurotoxin production [6]. This requires rigorous testing in food preparations considered at risk of botulism, to search for preformed BoNTs or BPC. BPC in food can be conveniently detected by PCR, with protocols targeted at BoNT-encoding genes, or through the mouse test, which can detect BoNTs in the supernatant of suitable enrichment media inoculated with the tested food sample [7]. A food sample is considered positive for BPC through the mouse test when the supernatant contains toxic substances that are lethal to mice, is heat-labile and neutralized in vivo by appropriate polyclonal antibodies raised against purified BoNTs of different serotypes [8]. When clinical signs consistent with botulism are displayed despite the use of specific antitoxins, the sample requires further investigation as botulism could be due to unknown BoNT serotypes.

A strict relative of BoNTs is tetanus neurotoxin (TeNT), produced by *Clostridium tetani*. TeNT is responsible for tetanus which is characterized by a spastic paralysis [9]. TeNT and BoNTs are very similar protein toxins, both in terms of structure and cellular mechanism of action [10,11], yet they display little homology in terms of amino acid composition. Such variability accounts for their different antigenicity.

Here we report the relevant observation that a TeNT produced by a *C. tetani* strain present in a food sample is neutralized by antisera raised against BoNT/B. The implications of this finding in the detection of BPC from food samples are discussed.

## 2. Results

The mouse test revealed the presence of a heat-labile neurotoxin neutralized by the trivalent A, B, E antitoxin and by two type-B monovalent antitoxins. Mice died between 10 and 24 h and the clinical signs were not observed; however, see below.

The PCR tests to detect BoNT genes (A to F) were negative.

**Figure 1:**
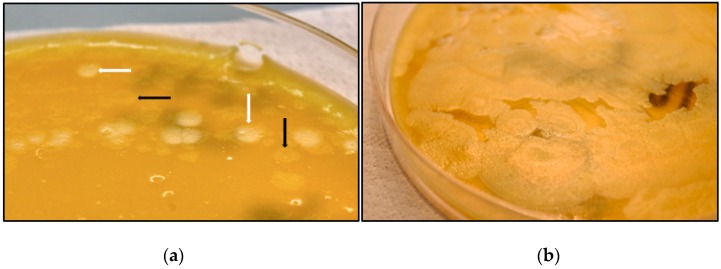
Macroscopic aspect of *Clostridium tetani* strain TV1277. (**a**) Lipase-positive (white arrows) and lipase-negative (black arrows) colonies in a 48 h-old pure culture (dimorphism). (**b**) After one week of incubation all colonies appear lipase-positive.

Bacteriological examination revealed the presence of lipase-positive and lipase-negative anaerobic colonies in EYA (Figure 1) and uniform, weakly haemolytic, rhizoid and swarming colonies in BAB2. Both lipase-positive and lipase-negative colonies were lecithinase-negative. These two types of colonies were sub-cultured on EYA and, 24 h later, the two subcultures appeared to be morphologically identical and lipase-negative. Forty-eight hours later, lipase-positive colonies appeared in both subcultures. One week later, all the colonies that appeared were lipase-positive (Figure 1).

**Figure 2:**
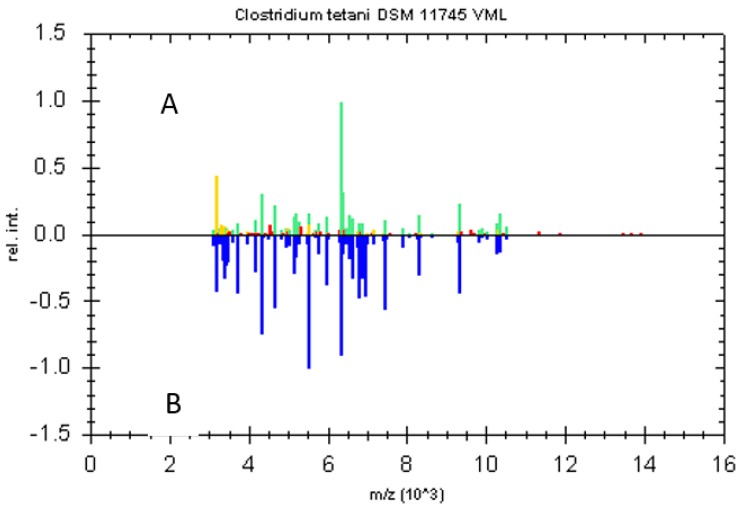
The peak list of the strain TV1277 spectrum is displayed in the upper half of the graphic. The color of the peaks reflects the degree of matching with the reference MSP (green = full match, yellow = partial match, red = no match). The lower half of the graphic displays the peak list of the reference MSP (*C. tetani* DSM 11745) in blue using an inverted intensity scale.

All the isolates were identified as *Clostridium tetani* by MALDI TOF MS with scores equal or higher than 2.489. The first best match of the strain was with the spectrum of the *C. tetani* reference strain DSM 11745 included in the default-database (Figure 2). This *C. tetani* strain was dubbed TV1277. We concluded that, for unknown reasons, there was an initial difference (dimorphism) in the appearance of the colonies cultured in EYA due to the lipase-reaction.

The mouse test was subsequently repeated with the supernatant obtained from TPGY containing a 4-day-old pure culture of the TV1277 strain. The results were the same as the ones described for the TPGY inoculated with the polenta sample but sternal recumbency, general paralysis and dyspnea were observed at 12 h post inoculation. No specific clinical signs referable to tetanus were noted. Postmortem examination revealed pulmonary lesions compatible with respiratory failure.

The neurotoxic effect of the tetanus neurotoxin (TeNT) produced by the pure culture of the *C. tetani* strain TV1277 was neutralized by 100 and 1000 UI/mL antitoxin type B (NIBSC), but not by 10 UI/mL. All the experiments in animals are summarized in Table 1.

**Figure 3:**
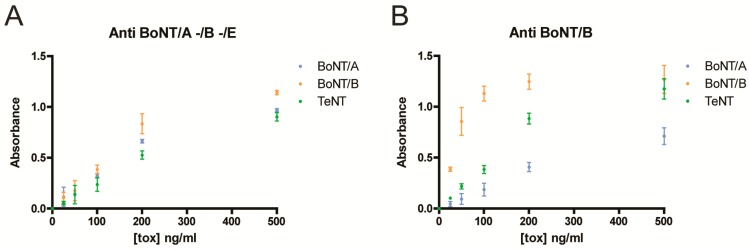
Cross reactivity of trivalent (**A**) and monovalent (**B**) BoNT-antitoxin with TeNT. Indicated concentrations of BoNT/A1 (cyan), BoNT/B1 (orange) and TeNT (green) were immobilized onto 96-well. Trivalent botulinum antitoxin (types A, B, E) from CDC (**A**) or monovalent antitoxin type B from the National Institute for Biological Standards and Control (NIBSC code: 60/001) (**B**) were used to assay immunoreactivity by indirect ELISA. Values reported were normalized subtracting background absorbance and are expressed as mean values of triplicates.

The ELISA showed that both trivalent (A, B, E) and monovalent (B) antitoxins reacted with TeNTs produced by the *C. tetani* reference strain (ATCC 10779) (Figure 3). Notably, trivalent antitoxin cross-reacts with TeNT to a similar extent with respect to BoNT/A1 and BoNT/B1. Monovalent antitoxin displays higher affinity for BoNT/B and sizeable cross activity with TeNT, that is practically equal with high toxin amount. A minimal cross reactivity with BoNT/A is also present.

## 3. Discussion

The present report describes the isolation of a *C. tetani* strain (TV1277) producing a neurotoxin that is neutralized by two different commercially available type B botulinum antitoxins in the mouse test.

Although *C. tetani* is not considered a food-borne pathogen, it can be present in foodstuff and interfere with the detection of BPC by the mouse test, considered the gold standard for BoNT detection from culture supernatants, giving rise to misleading results. 

The protocols for performing the mouse test specify that death without clinical signs is not adequate evidence that botulinum toxin was present in the injected material [12]. The mice should be observed for 4 days but death preceded by neurological signs can occur in the absence of animal facility personnel, as initially happened to us. Furthermore, clinical signs of tetanus and botulism can be indistinguishable in mice when a large amount of TeNT has been injected [13,14]. It is therefore advisable to perform the mouse assay with progressive dilutions of the sample until a concentration where the progression of the symptoms is obvious, but this may raise ethical issues.

We do not know if CDC antitoxins are produced in animals that are regularly vaccinated against tetanus neurotoxin (TeNT). However, NIBSC antitoxin B is produced in horses reported to be unvaccinated for tetanus [15]. Moreover, any interference from tetanus vaccine antibodies should be observed with all types of antitoxin tested, not just antitoxin type B.

**Figure 4:**
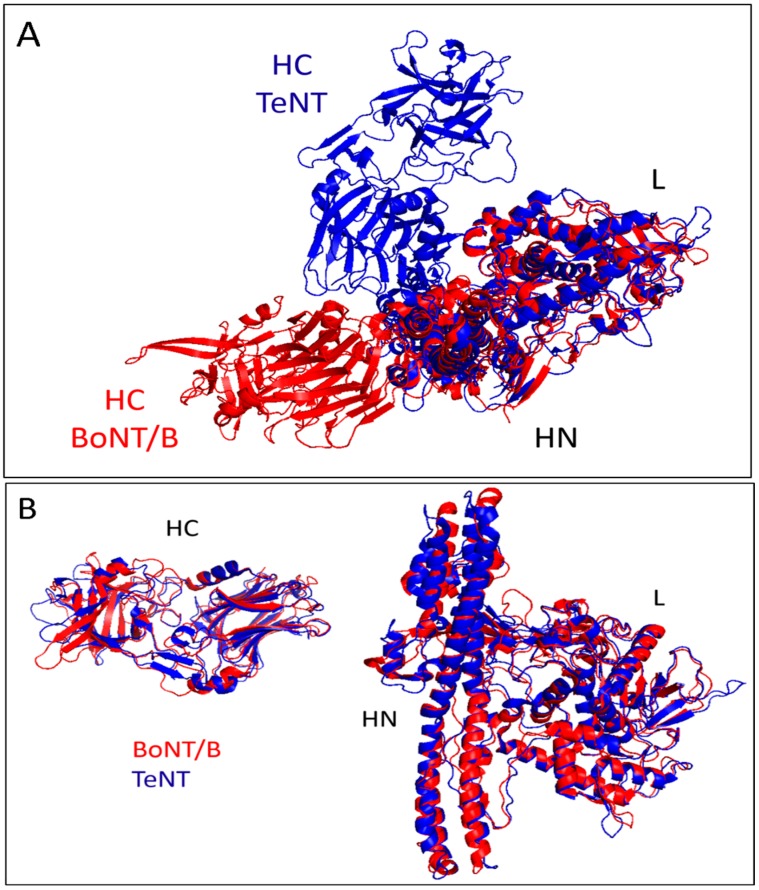
Spatial folding of tetanus (blue) and botulinum B (red) neurotoxins. (**A**) Notice the different position of the HC domain with respect to the other two domains (HN and L) in the two neurotoxins. (**B**) Overlapping of the two HC domain (**left**) and of the L-HN domains (**right**) of the two neurotoxins. The almost complete overlapping of the folding of the alpha-carbon chains of the two toxins supports the possibility of the existence of common antigenic determinants in the two toxins.

One explanation for this “off-target” neutralization of the TeNT effect with botulinum type B antitoxin might be the existence of common epitopes between these two neurotoxins. This possibility is reinforced by some findings reporting that TeNT shares common antigenic sites with BoNTs types B, C, D and E, recognized by specific monoclonal antibodies [16]. However, in the present study, only monovalent type B antitoxin showed an in vivo neutralizing capability towards the TeNT produced by strain TV1277. Furthermore, BoNTs and TeNT exhibit a very similar folding of the three domains composing their structure, making it very likely that some sequential and conformational epitopes of BoNT/B may be present in TeNT [2]. This conclusion is further supported by the close overlapping of the alpha-carbon chain folding of BoNT/B (in red) and that of TeNT (in blue) showed in Figure 4 [11]. Panel 2A shows the similar three-domain structure of the two toxins with the different spatial position of their binding domains HC with respect to domains L and HN. Panel 2B shows that when the single HC and L-HN domains of TeNT (blue trace) and BoNT/B (red trace) are overlapped a substantial similarity is found. Clearly, as antigenic sites are determined also by the lateral amino acid chains, this superimposition should be analyzed further. However, the overall similar folding speaks in favor of the possibility that common antigenic determinants, playing a major role in the neuronal paralysis induced by these potent neurotoxins, do exist.

Despite the above common characteristics, a cross-neutralization effect of TeNT in vivo due to botulinum antitoxin type B has never, to the best of our knowledge, been reported before.

In future studies we will investigate whether this “in vivo” neutralizing effect of botulinum antitoxin B is observed only for the TeNT produced by strain TV1277 or if it is a common characteristic of TeNTs produced by different strains. The results of the ELISA test obtained with the TeNT produced by the Harvard E88 strain support the second hypothesis. In addition, the amino acid sequence of TV1277 TeNT revealed only 17 mutations out of 1315 amino acids of the reference TeNT produced by the Harvard E88 strain (data not shown).

Curiously, the lipase-positive reaction of strain TV1277 contrasts with the published biochemical properties of *C. tetani* [17], but is characteristic of *C. botulinum* groups I, II and III [18]. We subsequently repeated isolation of lipase-different colonies three times and, after one week, all the subcultures showed a uniform, lipase-positive reaction.

PCR proved to be a reliable tool that should replace the mouse test for BPC detection, partly for ethical considerations. However, this method is instead inadequate for detecting unknown BoNT-encoding genes.

The presence of TeNTs that react with botulinum antitoxin might also interfere with immune-enzymatic methods for detecting BoNTs in culture supernatants. Accordingly, these kits should additionally be validated for specificity towards such TeNTs.

Humans are not usually vaccinated against BoNT type B but citizens in many countries are regularly vaccinated for tetanus. It might be interesting to investigate whether the human vaccine for type B botulism also protects from certain isoforms of TeNTs. It is possible that people vaccinated for type B botulism could be simultaneously protected against tetanus.

In conclusion, our results demonstrate that *C. tetani* may present in foodstuffs and can interfere with the detection of BPC by the mouse bioassay, considered the gold standard for detecting BoNT in culture supernatants.

## 4. Materials and Methods

### 4.1. Detection of Botulinum Neurotoxin-Producing Clostridia by the Mouse Bioassay

In December 2016, a food producer located in the province of Trento prepared a batch of vacuum-packed polenta, a maize porridge preparation typical of the North of Italy. The polenta was vacuum-packed in glass jars and one aliquot of the stock was sent to the laboratory for the routine microbiology testing that precedes consumption of the product.

The sample was screened for the presence of BPC through the mouse bioassay (or mouse test) as previously described [8,12]. Briefly, 25 g of the sample were introduced into 225 mL of trypticase peptone-glucose yeast extract (TPGY), heated to 80 °C for 10 min and incubated at 30 °C for 4 days [19]. The supernatant was collected, centrifuged at 8000× *g* and filtered with a 0.45 µm filter (Millipore, Tullagreen, Ireland). Two mice were intraperitoneally injected with 0.4 mL of the untreated supernatant and two with 0.5 mL of the supernatant previously heated to 100 °C for 10 min. Next, 0.25 mL of the trivalent botulinum antitoxin (types A, B, E) supplied by the Center for Disease Control (CDC) was mixed with 1 mL of supernatant and, after 45 min, 0.5 mL were intraperitoneally injected into two mice. This step was then repeated with monovalent CDC antitoxins types A, B, C, D, E, F and titrated monovalent antitoxin type B supplied by the National Institute for Biological Standards and Control (NIBSC, code: 60/001). Mice were injected at 9.00 a.m. and observed at 4, 6, 8, 10 and 24 h post inoculation. The surviving mice were monitored for a further 48 h.

The mouse test was then repeated with the supernatant obtained from TPGY containing a 4-day-old pure neurotoxic culture isolated as reported in paragraph 2.3. The NIBSC antitoxin was used in this supernatant at 10, 100 and 1000 UI/mL to determine the neutralization titer after inoculation of two mice for each dilution. A volume of 0.25 mL of tetanus antitoxin (NIBSC, 60/113) containing 10 UI/mL, was mixed with 1 mL of the filtered supernatant of a 4-day-old neurotoxic culture. Forty-five minutes later, 0.5 mL were inoculated intraperitoneally in two mice.

Dead mice underwent postmortem examination.

### 4.2. PCR Protocols for Detecting and Characterizing BoNT-Encoding Genes

After 48 h of incubation, 175 μL of the inoculated TPGY broth were collected from the bottom of the tube and DNA was automatically extracted (Microlab Starlet, Hamilton, Bonaduz, Switzerland) using the MagMax Total Nucleic Acid Isolation kit (Ambion/Life Technologies, Carsland, CA, USA). Real-time PCR and PCR methods for *C. botulinum* neurotoxin genes types A to F were applied in accordance with previously published protocols [20,21].

### 4.3. Bacteriological Examination

Fifty microliters of TPGY were plated on egg yolk agar (EYA) produced as previously described [22] and incubated for 48 h at 37 °C in an anaerobic cabinet (Shel Lab, Cornelius, OR, USA) with an atmosphere composed of 5% hydrogen, 5% carbon dioxide and 90% nitrogen. Single colonies with different macroscopic morphology and/or different lipase/lecithinase reactions, were collected and streaked on 2 different plates of Blood Agar Base No.2 (BAB2) (Oxoid, Hampshire, UK) and 2 plates of EYA. One plate for each medium was incubated in aerobic and one in anaerobic conditions at 37 °C for 48 h. Colonies growing only in anaerobic conditions were then identified by MALDI TOF MS (Biotyper Microflex LT, Bruker Daltonics, Bremen, Germany), using the MALDI Biotyper software package (version 3.0, Bruker Daltonics, Bremen, Germany) and an “in house” database created with *C. botulinum* reference and field strains [23]. As specified by the manufacturer, a score value of <1.7 indicated that identification was unreliable; scores between 1.7 and 2.0 that identification was reliable at the genus level; scores between 2.0 and 2.3 that it was reliable at the genus level and probable at the species level; scores higher than 2.3 indicated highly probable species identification.

### 4.4. ELISA Assay

Ninety six-well polystyrene plates (Sarstedt, Nümbrecht, Germany) were coated with 100 µL of BoNT/A1, BoNT/B1 or TeNT (E88 strain) solutions at the concentrations: 25, 50, 100, 150 and 500 ng/mL diluted with PBS and incubated overnight at 4 °C [24]. Toxins were purified as described previously [23]. Thereafter, wells were treated with 2% BSA for 1 h, washed with PBS 0.05% Tween20 (PBST) and incubated for 2 h at room temperature with indicated antitoxins (CDC) diluted 1:200 in PBST supplemented with 0.1% BSA (Sigma Aldrich, St. Louis, MI, USA). Samples were then extensively washed with PBST and incubated 1 h with HRP-conjugated secondary antibody. After washing, 100 µL of 2,2′-Azino-bis(3-ethylbenzothiazoline-6-sulfonic acid) (ABTS) was added and absorbance read at 405 nm with a microplate reader. Values reported were normalized for the absorbance of the wells coated without toxins.

### 4.5. Experiments on Animals

The mouse test was conducted in accordance with Italian and European legislation on ethical standards (European Communities Council Directive [2010/63/EU] on the protection of animals used for scientific purposes).

The mouse test was approved by the Ethics Committee of the Istituto Zooprofilattico Sperimentale delle Venezie (opinion No. 24/2014) and officially authorized by the Italian Ministry of Health (authorization No. 239/2015-PR) on 9 April 2015.

## Figures and Tables

**Table 1:** Experiments in animals with filtrated supernatant of a 4-day-old culture of *C. tetani* TV1277. For each experiment, two mice were injected intraperitoneally.

Antitoxin	Producer	Antitoxin Titre	Number of Dead Mice at 24, 48 and 72 h Post Inoculation		
			24 h	48 h	72 h
Botulinum trivalent antitoxin A, B, E	CDC	>10 UI/mL	0	0	0
Botulinum antitoxin A	CDC	>10 UI/mL	2	-	-
Botulinum antitoxin B	CDC	>10 UI/mL	0	0	0
Botulinum antitoxin C	CDC	>10 UI/mL	2	-	-
Botulinum antitoxin D	CDC	>10 UI/mL	2	-	-
Botulinum antitoxin E	CDC	≥10 UI/mL	2	-	-
Botulinum antitoxin F	CDC	≥10 UI/mL	2	-	-
Botulinum antitoxin B	NIBSC	10 UI/mL	1	1	-
Botulinum antitoxin B	NIBSC	100 UI/mL	0	0	0
Botulinum antitoxin B	NIBSC	1000 UI/mL	0	0	0
Tetanus antitoxin	NIBSC	10 UI/mL	0	0	0
**Further samples tested**					
Untreated supernatant	-	-	2	-	-
Heat treated supernatant	-	-	0	0	0
